# Cultural Attitudes Toward Weight, Diet, and Physical Activity Among Overweight African American Girls

**Published:** 2008-03-15

**Authors:** Josephine E. A. Boyington, Lori Carter-Edwards, Mark Piehl, Jeanne Hutson, Debbe Langdon, Shilpa McManus

**Affiliations:** Institute for Health, Social and Community Research, Shaw University. Boyington is also a diversity supplement research investigator, National Institute of Arthritis and Musculoskeletal and Skin Diseases, University of North Carolina School of Medicine, Thurston Arthritis Research Center, Chapel Hill, North Carolina.; Health Promotion and Disease Prevention, Division of Community Health, Department of Community and Family Medicine, Duke University, Durham, North Carolina; Pediatric Diabetes Program, WakeMed Health and Hospitals, Raleigh, North Carolina, and Clinical Associate Professor of Pediatrics, University of North Carolina School of Medicine, Chapel Hill, North Carolina; Pediatric Diabetes Program, WakeMed Health and Hospitals, Raleigh, North Carolina; Pediatric Diabetes and Asthma Programs, WakeMed Health and Hospitals, Raleigh, North Carolina; University of North Carolina School of Medicine, Chapel Hill, North Carolina

## Abstract

**Introduction:**

The growing epidemic of childhood obesity has led to an increasing focus on strategies for prevention. However, little is known about attitudes and perceptions toward weight, diet, and physical activity among American youth, and particularly among young African American females. This pilot study sought to qualitatively explore cultural attitudes and perceptions toward body image, food, and physical activity among a sample of overweight African American girls.

**Methods:**

We recruited 12 overweight girls, aged 12 to 18 years, from a hospital-based pediatric diabetes screening and prevention program. Five semistructured group interviews were conducted to explore attitudes on weight, diet, and physical activity. Sessions averaged 1 hour and were conducted by trained interviewers. Data were transcribed and evaluated for content and relevant themes.

**Results:**

The following themes emerged: weight and body size preferences were primarily determined by the individual and her immediate social circle and were less influenced by opinions of those outside of the social circle; food choices depended on texture, taste, appearance, and context more than on nutritional value; engagement in recreational physical activity was influenced by time constraints from school and extracurricular activities and by neighborhood safety; participation in structured exercise was limited because of the cost and time related to maintenance of personal aesthetics (hair and nails); and celebrities were not perceived as role models for diet and physical activity habits.

**Conclusion:**

In this sample of girls, the findings imply that perceptions of weight and healthy lifestyle behaviors are largely determined by environmental and personal influences. These factors should be considered in the development of healthy-weight interventions for African American girls.

## Introduction

Childhood overweight has reached epidemic proportions in the United States. For the years 1999 through 2002, African American females aged 12 to 19 years had the highest prevalence of overweight (23.6%) compared with white (12.7%) and Mexican American girls (19.9%) (1). Overweight is defined as body mass index greater than 95th percentile for age.

Poor nutrition and a sedentary lifestyle are key determinants of excess weight among all U.S. children (2). Compared with young white females, young African American females are less likely to eat the recommended daily amount of fruits, vegetables, and whole grains, and are more likely to consume high amounts of fat and sodium (2,3). They also are less likely to be physically active and more likely to watch television, and to watch television while eating (4,5). Moreover, African American females prefer and tolerate heavier body weight and are more satisfied with their body image and larger body size (6,7). Consequently, they maintain greater within-group social acceptance of heavier body weight and perceive less social pressure to lose weight (8-11), resulting in infrequent pursuit of long-term weight management strategies (12,13). These findings are of concern because excess weight in childhood is associated with a dramatically increased risk of adult obesity and obesity-related conditions such as diabetes and cardiovascular disease (14-16). Furthermore, excess weight in adolescence is associated with poorer social and economic outcomes in adulthood (17,18).

In light of these findings, national health agendas target lifestyle modification as a first-line prevention strategy for overweight and its complications and encourage further understanding of barriers to lifestyle modification among minority populations (19).

Thomas (20) posits that lifestyle behaviors of African American females are responses to historical, social, and cultural forces affecting their personal health beliefs, attitudes, and perceptions. Though unhealthy diet and physical inactivity are clearly determinants of excess weight in this group, few data exist on the psychosocial factors (beliefs, attitudes, and perceptions) that promote these behaviors and the cultural contexts within which they operate (21).

Culture, defined as the unique shared values, beliefs, and practices of a group, can influence the behaviors of individuals by affecting their thoughts, feelings, acceptance, and adoption of health education messages (22,23). For example, to reduce the prevalence of excess weight, current health strategies promote lower fat intake. However, among African American populations, especially in the southeastern United States, consumption of foods high in fat is a documented dietary and cultural practice (24). Nationally, compared with young white females, young African American females consume more calories from fat and report that social influences have a greater impact on their dietary practices (25). However, knowledge of how the culturally based perceptions and attitudes of African American youths are related to excess weight (26) and specifically how caregivers influence dietary and physical activity choices of youths remains limited (26,27). Among a sample of African Americans, one study found that most caregivers of overweight children aged 5 to 10 years tolerated larger child body sizes and minimized their child's health risk (27). Another study reported that African American girls perceive female caregivers as important role models for body size ideals (28), indicating the importance of assessing caregivers' influence on weight-related attitudes and behaviors of youths.

Further investigation of these social and environmental influences may help explain why this population is at disproportionate risk for obesity and obesity-related conditions and may lead to innovative strategies for encouraging the adoption of healthy lifestyle changes. This pilot study sought to qualitatively examine perceptions, attitudes, and cultural experiences relative to weight and weight-related factors among a sample of overweight African American girls.

## Methods

### Sample

This predominantly low-income sample consisted of mother-daughter dyads recruited from a large urban hospital diabetes prevention program in North Carolina during October 2005 through July 2006. Girls were eligible if they were screened as overweight and were part of the hospital diabetes prevention program but had not been diagnosed with diabetes, prediabetes, or metabolic syndrome. To assess the perceptions of both mothers of overweight girls and girls themselves, dyads were recruited and interviewed concurrently but separately. Of the 49 eligible mother-daughter dyads, we successfully recruited 30, and 12 completed the study. Barriers for noncompletion included competing priorities that prevented attendance to scheduled sessions; unreliable phone services because of the inability to pay; and frequent address changes because of participants' relocation.

### Procedures

Supervised student interns used clinic records to recruit eligible dyads by phone. At initial contact, each mother received the full study description and dual invitations to participate with her daughter in a concurrent but separate interview session. Girls were matched by age group (12–14 years and 15–18 years), and mothers by age of daughter, into one of ten weekday sessions held at the hospital facilities. Five of the sessions targeted girls and five targeted mothers. Data presented here represent the findings from the girls' sessions only.

Before each recorded session, we obtained dual consent and assent from each dyad. At completion, a $30 gift card was given to each pair. The study protocol was approved by the institutional review boards of Shaw University, the University of North Carolina (UNC) at Chapel Hill, and WakeMed Health and Hospitals. The study was funded by the UNC Program on Ethnicity, Culture, and Health Outcomes (ECHO) and the Carolina-Shaw Partnership for the Elimination of Health Disparities (through the Centers of Excellence Partnerships for Community Outreach, Research on Health Disparities and Training Project [Project EXPORT]), which is supported by the National Center on Minority Health and Health Disparities. Funding for the diabetes screening program, from which we recruited participants, was provided by the John Rex Endowment, Raleigh, North Carolina, as part of a larger community-wide diabetes prevention effort.

### Data collection and analysis

The youth interview guide was designed to elicit perceptions and attitudes toward variables related to excess weight. Discussion topics were informed by the literature and by the investigators' understanding of the interrelationship of factors that contribute to overweight. We assessed six key areas: 1) definition of personal health; 2) dietary and physical activity habits; 3) perceptions of personal health and body; 4) important figures and role models; 5) social support; and 6) culture and environmental factors.

Trained interviewers conducted and recorded semistructured interviews. Transcribed text was verified for content accuracy by the lead investigator, who compared the transcripts against the recordings and coded them using NVivo 7 software (QSR International, Doncaster, Victoria, Australia). Coded transcripts were then reviewed by the second author and another evaluator. In cases of differences in coding, the third evaluator served as referee. Following the method described by Krueger (29), matrices of codes and corresponding quotes were developed and used with the raw data to explore for areas of divergence and convergence. To ensure validity of findings, we organized emergent themes by domains, mapped according to research questions, and checked across groups for frequency of occurrence.

## Results

### Weight and body size perceptions

For more than three-quarters of the participants, having excess weight began in early childhood and was associated with negative comments from peers. However, regardless of age, the girls stated that they were "conditioned" against hurtful, weight-related comments and were neither bothered by them nor motivated to make substantial changes to their nutrition and physical activity habits. They perceived that "being skinny" was not in their best interest:

When I was in elementary school people called me fat. . . . I be like, okay, and, "Are you finished?" They be like, "Man, shut up." I say, "Dag," 'cause you know I don't care . . . 'cause to me it's like words are words, they go through one ear and out the other.

I've felt like they can call me the names they want to, but . . . being like really skinny is not good either.

Though clinically screened and categorized as overweight, the girls described their body size using a culturally based normative scale anchored by "skinny" and "big." Other descriptors in the scale included "medium," "thick," "fat," and occasionally "obese." Rarely was weight expressed as an objective measure:

I'm like, "Am I big?" They're like, "no." Then I was like, "Then what category I am? Skinny, big, or fat?" I don't like skinny, and she's like, "You ain't in the fat category, you ain't in the skinny, but you ain't all the way big. You going straight."

Instead of an ideal weight or body size, the girls preferred a continuum of acceptable sizes. They perceived that self-satisfaction with size was more important than actual size. Hence, a healthy body size was one with which an individual felt "comfortable": "I think being comfortable is just healthy. Being comfortable with your weight."

Preferred body size was perceived to be a personally determined attribute, yet the reference for body size was their families and cultural group. Weight and body size were, therefore, socially comparative attributes but were individualized through the girls' personal perceptions of their own image:

My family, they . . . compare me to my other cousin. That she's got like a perfect body, like a very perfect body, and they compare me to her like, "One day you're gonna look [like] her," and then my momma be like, "No, she's gonna look like her sister."

In response to the interviewer's question to define a healthy weight, one participant responded with this quote, to which others assented:

Like not past 200. . . . That's what they said when I went to the diabetic clinic. They want [me] to be like 150 and 132. I was like 150 when I was in 5th grade. If I go back to 5th grade I'll try to maintain that.

Among those who verbalized understanding that weight is an objective measure and related it to health outcomes, there was also a perception that their overweight status was not "critical." Following objective National Heart, Lung, and Blood Institute guidelines (30), however, the girls perceived "obesity" to be the critical stage:

I know I'm not healthy. . . . I am not like overweight. . . . I am overweight. I am not like at the obesity stage yet. And I don't think I'm going to be in the obesity stage.

Consistently, preferred body size was described as one that was large, especially in the breasts and buttocks area. Large breasts and buttocks were perceived as attributes of physical attractiveness; when these attributes were lacking, it resulted in dissatisfaction and led to remedial actions:

They be complaining. I got one friend she be complaining, she be like, "I wish I had a big butt."

I got like two [friends] and they real skinny, but N, she got little boobs [breasts], and T, she be stuffing her bra . . . she be trying to wear jeans to make her butt big.

Further illustrating that larger body size can be a social asset and not a hindrance, one participant mentioned a physically large television actress and commented on her off-screen life:

"Nicky Parker" [a physically large television character] — She funny and she don't care what nobody say. . . . Well, she got a husband [in real life]. It proves that you can be big and have nice people, nice things, 'cause her husband, I see him . . . he's nice looking.

### Dietary perceptions

The girls reported that their selection and consumption of healthy foods depended foremost on the taste, texture, and appearance:

It's how it's cooked. . . . If my grandma cooks something nasty, like what she wants . . . she will cook me something else or I make me something else. . . . Like today I'm like, "Can I have some pizza?" And she would say, "Okay." . . . Like at breakfast my grandma will cook, but I don't want to eat because I don't like grits — it's nasty to me.

They told us that the school food is healthy for you because they "shake and bake." And there is no seasoning [in] the mashed potato, there's no flavor. They be kicking 'cause you don't want it.

The participants emphasized that in addition to taste, dietary decisions also included considerations of context. Family and peer considerations often superseded personal judgment to eat healthfully as the girls strove to preserve cohesion in various social contexts:

And like who's going to pass up their grandma's cooking? She's cooking like Sunday, and it be like she's cooking chicken, collard greens, corn bread, and you know it's your grandma. She's old-fashioned so you don't . . . you be like, "Grandma, can you kind of cut back on the grease?", and she's going to look at you like, "Are you crazy?"

Evidencing their internal struggle in balancing healthy eating habits in various contexts, the girls specifically stated they needed self-management skills: "I need to learn how to say 'no.' "; "I need more willpower."

Limited support for making healthy food choices at school was noted as a key hindrance: "At school . . . they offer you the opportunity to get fruit but they don't emphasize, okay, well, we want you to eat this and this and this."

Healthy foods were not generally perceived as filling. Though the girls identified the nutrition habits of their white schoolmates as healthy, they avoided pursuit of this dietary behavior because they perceived lack of physical satiety as a hindrance:

[White girls] eat nutrient bars, or they bring their lunch, have their little Juicy Juice and granola bars. I know 'cause . . . I watch them eat lunch and I be like, "How do you all get full?" There's nothing there.

They'll have this little wheat bread sandwich, that's all. They eat an apple, and a drink. . . . It's like Lunchables.

The girls identified culture as a major influence on the dietary decisions of their white schoolmates and specifically stated that it had a polarizing impact. On one hand, the African American girls thought that the diets of their white schoolmates promoted healthy eating, but on the other hand they perceived such a diet to negatively affect white girls' self-esteem. "I guess [the white girls] are raised to be [that way]."; "[White girls] care about what they look like. . . . They care about what people think. Like if they are too skinny they'll go home and cry. They have low self-esteem." Many of the African American girls indicated by this and similar other comments that they valued physical satiety over other people's expectations and opinions.

Traditional African American foods and food culture were identified as determinants of current nutrition behaviors. However, the girls noted that traditional foods were not inherently unhealthy but were made so by the preparation methods:

Well, other races, they be trying to eat healthy. . . . Asians, white people be, they like, they have . . . salad right there . . . then the main course, and they don't use all the fat in it, or they'll go out and eat nice foods.

### Physical activity perceptions

The girls participated less enthusiastically in discussion of exercise than in other discussion segments. They reported a preference for group physical activities, such as step and dance teams and basketball. However, their reported frequency of participation varied because of the perceived "beauty cost," perceived lack of time, and reported lack of access to preferred activities. In discussing the "beauty cost," they externalized their comments and spoke about "others," indicating this attitude was not perceived to be a problem for them:

[Girls are] not interested in working like real hard doing all that exercise stuff. Some of them like — like girls they don't like to sweat and get their hair messed up. . . . They think like they do the wrong thing, they break their nails, it's a crisis.

Participants also complained about not being able to participate in preferred, school-sponsored physical activities. They specifically cited their inability to meet qualifying prerequisites for weight and blood pressure as hindrances. They perceived their current physical activity options as limited because 1) more people wanted to participate in offered activities than could be accommodated or 2) desired activities were not offered at their schools. They also thought exercise was not a culturally valued practice and perceived it as a low-ranking, optional behavior that made demands on their limited time and resources: "I'll do it later."

As with other factors, physical activity was often discussed in the context of school, neighborhood, and family environments. The lack of safety after school was cited most often as a physical environmental factor that negatively influenced physical activity habits:

I think there's no way for me to exercise unless I go to school for PE or health [class] 'cause I don't have a good neighborhood to exercise or have fun with anybody around our neighborhood.

My neighborhood is real bad. . . . It's like real lonely. Well, not all the time, like other kids have groups they hang out with, and like you'd be scared to be out at night.

### Important figures and role models

Most participants acknowledged that adult females were their role models and influenced their eating habits through food purchasing and preparation. However, the girls resisted verbal direction related to improving nutrition and physical activity from these adults because the girls wanted to determine their own approach. Where the need for support was perceived, the participants reported positive verbal support as enabling positive behaviors or buffering negative behaviors, or both. With the exception of three girls who mentioned a grandfather or father, the influence of older males as a source of support for good dietary and physical activity decisions was absent in the discussions. From male peers, the girls reported receipt of negative support in the form of comments directed toward their body parts. For example, one girl relayed how a friend was teased by a male peer that she had "mosquito bites" for breasts. So, despite previously stating that they were "conditioned" against the effect of negative comments, the girls seemed to be affected by male peer teasing to the extent that one said, "He lowers my self-esteem."

An unanticipated finding was that some celebrities who speak on weight-related issues were not perceived to be genuine and, therefore, not emulated. Incentives such as financial compensations and access to personal gyms, trainers, and chefs were perceived as factors that lessened the relevance of the celebrities' messages: "[Celebrities] get paid to say what they say. . . . Toni Braxton, you know how she been big, she got her own private instructor"; "[Oprah Winfrey], she got her own gym in her house."

## Discussion

To address the high prevalence of overweight among youth, investigators have called for consideration of youths' perceptions (26,31) in addition to other factors. This pilot study explored and identified important weight-related perceptions and attitudes among a sample of overweight African American girls. Pertinent findings were that 1) weight and body size preferences are personally determined attributes that are less influenced by the opinions of those outside of one's social circle; 2) food choices depend on texture, taste, appearance, and context of the food; 3) engagement in physical activity is influenced by social and environmental factors such as time constraints imposed by school and extracurricular activities; 4) participation in structured physical activity is limited because of the cost in time related to maintenance of personal aesthetics (hair and nails); and 5) some celebrities are not realistic models for diet and physical activity habits. In this sample of girls, these findings suggest that weight-related behaviors and attitudes are influenced by culturally based perceptions as well as contexts.

Previous studies of African American girls document a preference for larger body size, which is often supported by positive reinforcement from adults (5,6). This study further adds that body size was perceived to be related to a specific stage of life. In their discussions, participants specifically contrasted their weight in elementary school with their current weight and noted that weight was stage-dependent, implying that as one matures in age, one is supposed to progress to a larger body size and weight. Additionally, as with previous findings in adult females, the girls in this study conceptualized body size and weight on a normative, culturally based scale (32) and mentioned family and friends as their body size references. This finding suggests that weight and body size are socially comparative attributes in this sample and further illustrates how culture affects girls' values and their self-estimations of their bodies.

Personally oriented values such as physical attractiveness, personal satisfaction, self-esteem, and comfort were consistently expressed by participants in their descriptions of body size, dietary preferences, and thoughts about other cultural groups. For example, nutrition and weight habits of white girls were perceived to be externally motivated and the cause and evidence of low self-esteem, therefore, an undesirable behavior. The participants stated they valued physical satiety over others' opinions and consequently evidenced personal responsibility for their own healthy sense of self. Incidentally, studies comparing black and white women report that despite experiencing comparable displeasure at negative comments, black women's self-esteem is not influenced by general positive or negative social feedback (33). In addition, Patterson (34) posits that among African American females, relevant "others" serve as the source of self-estimations and enable them to be rooted against negative self-estimations (34). Because the girls in our study indicated that the relevant people in their lives were their African American friends and families, these people could be integral to the success of efforts targeting weight reduction in this population.

The physical characteristics of food and contextual challenges related to dietary choices were mentioned as hindrances in the pursuit of healthy nutrition habits. Participants had adequate knowledge of healthy nutrition but perceived a lack of self-management and negotiation skills. Their expressed desire to acquire these skills indicated a willingness to take responsibility for their choices and suggested that efforts focusing on self-efficacy may be highly beneficial in this sample of girls.

Limitations to engagement in physical activity included the amount of time already taken up with school and extracurricular activities, limited access to opportunities for exercise, time and beauty cost, and lack of safety. Collectively, these challenges signal interplay between personal factors and global societal factors over which these girls lack control. Furthermore, the challenges suggest potential areas for creative explorations for public health interventions. For example, approaches favoring school- or home-based physical activities have not been well explored in this population and may be useful for these girls, who perceived lack of opportunities because of limited access to activities and because of unsafe neighborhoods. Furthermore, concerns about personal aesthetics indicate that activities perceived as less disruptive might be more easily adopted than those perceived to be aesthetically costly.

The negligible impact of celebrities on diet and physical activity behaviors was an unexpected finding. Specific African American celebrities discussed were Oprah Winfrey, Toni Braxton, and Mo'Nique ("Nicky Parker"). Although the girls admired and mentioned these women because of their past or current weight issues, the perceived impact of these women varied. Weight-related messages from Oprah Winfrey and Toni Braxton were seen as irrelevant because the women were perceived to have assets not available to the girls. In contrast, Mo'Nique, the largest of the three celebrities, who embraces and positively promotes her large size, was admired and perceived as a realistic role model and a testament that one can be successful despite one's large size. For these girls, Mo'Nique may be a more relevant "other" because her attitude is in accord with their cultural values of weight and body size. Moreover, her self-affirmations coupled with her personal success also affirm their expressed desire to be responsible for their own healthy internal and external sense of self. These findings are informative because they highlight that public health interventions must consider both the delivery and the content of health messages targeting these girls. Hence, efforts that tap into personal values and enhance self-esteem may be more favorably perceived and potentially more relevant than celebrity-endorsed messages not reflecting the realities of the girls' daily lives.

One major determinant of chronic diseases is the environment and its influence on lifestyle behaviors (30). In North Carolina, African Americans are poorer, underemployed, more sedentary, and consume less than the recommended intake of fruits and vegetables than do whites (35). This environmental context and the behaviors reported in this study suggest that this study's findings may differ from findings in other regions of the United States.

Though the small sample size limits the study's generalizability, the perceptions and attitudes documented point to key areas for further inquiry. Overall, the findings are informative and suggest that future intervention efforts should assess girls' knowledge, perceptions, and self-efficacy levels related to nutrition and physical activity to inform program design. Assessments should specifically target identifying girls' perceptions of context-specific barriers (availability, cost, access, safety, health status) and facilitators (preferred foods, activities, role models) of adopting healthy behaviors. Furthermore, program effectiveness and sustainability can be enhanced by engaging, as change facilitators, role models who influence girls' weight-related perceptions and behaviors. Finally, program messages and offerings should be context-specific to promote ease of adoption and sustainability in this population.
